# Mental health professionals’ perspective on the use of esketamine in treatment-resistant depression and their motivation to adopt it: a Saudi cross-sectional study

**DOI:** 10.3389/fpsyt.2026.1726411

**Published:** 2026-02-27

**Authors:** Ahmad H. Almadani, Ayedh H. Alghamdi, Gosay M. Almazyad, Saleh I. Alfawaz, Mohammed A. Ghaith, Ali A. Alshehri, Mohammed A. Aljaffer, Saleh A. Alghamdi

**Affiliations:** 1 Department of Psychiatry, College of Medicine, King Saud University, Riyadh, Saudi Arabia; 2 Department of Psychiatry, King Saud University Medical City, King Saud University, Riyadh, Saudi Arabia; 3 Medical Department, Bupa CareConnect, Bupa Arabia, Jeddah, Saudi Arabia; 4 Psychiatry Department, College of Medicine, Imam Mohammad Ibn Saud Islamic University (IMSIU), Riyadh, Saudi Arabia

**Keywords:** attitudes, esketamine, physician motivation adoption scale, psychiatrists, Saudi Arabia, treatment-resistant depression

## Abstract

**Background:**

Esketamine is an innovative treatment for individuals with treatment-resistant depression (TRD). However, its adoption could depend on the perceptions and motivations of prescribing psychiatrists, among other factors.

**Objective:**

This study aims to explore the attitudes of psychiatrists (of all levels, including those in training) across Saudi Arabia toward the use of esketamine for TRD and investigate the motivational factors related to their willingness to adopt it.

**Methods:**

This is a cross-sectional study that utilized a convenience sampling method. The study tool consisted of a questionnaire developed by the research team and the Physician-Motivation Adoption Scale.

**Results:**

Of the 223 participants surveyed, 19.73% reported having prescribed esketamine, most commonly to 1–2 patients. The most frequently perceived adverse effects were dissociation/delusions/hallucinations (66.82%), followed by dizziness/vertigo (59.19%). Esketamine prescription was significantly associated with older age (p = 0.049), consultant-level practice (p = 0.003), practice in the Western Region (p < 0.001), lower concern about potential misuse (p = 0.027), perceiving easier access (p = 0.004), and fewer concerns about the administration process (p = 0.007). Ordinal logistic regression demonstrated that senior registrars, registrars, and residents were significantly less likely to prescribe esketamine than consultants (OR = 0.21, 0.17, and 0.10, respectively). “Not being sure” that cost was a barrier was associated with lower odds of prescribing (OR = 0.32, p = 0.022), whereas higher functional subscale scores were strongly associated with willingness to prescribe (OR = 1.62, p < 0.001). The most commonly reported barriers to prescribing esketamine included the administration process (66.37%) and cost (65.02%).

**Conclusion:**

Despite significant interest, actual adoption of esketamine is low, mainly due to logistical barriers, high costs, and availability issues. Concerns about side effects and potential misuse are also linked to hesitation. Accordingly, interventions to address the obstacles and concerns are needed.

## Introduction

Major depressive disorder (MDD) continues to be one of the most common and debilitating mental health conditions globally. The World Health Organization reported that more than 264 million people are affected worldwide, and MDD is projected to be one of the leading contributors to disease burden by 2030 (1, 2). In Saudi Arabia, national data suggest that approximately 6% of the population will experience MDD in their lifetime, revealing the serious public health implications of this illness (3). Although many people respond to first-line antidepressants, many fail to achieve significant improvement (reported to be 10%–30% of patients) after multiple treatment attempts (4). This clinical scenario is known as treatment-resistant depression (TRD), and it is difficult for both patients and physicians. Patients with TRD often live with ongoing symptoms, lower quality of life, and increased risk of suicide and relapse (4–6). Despite the broad range of treatment options, clinical outcomes for TRD remain inconsistent, and side effects often limit tolerability (5, 7).

Esketamine, a nasal spray made from the S-enantiomer of ketamine, is a new treatment option for TRD. It has been approved as a treatment for TRD by the United States Food and Drug Administration (FDA) and European Medicines Agency (EMA). It has a rapid-acting mechanism of action, which is thought to be through glutamatergic modulation and increased neuroplasticity, in contrast to traditional antidepressants (8–10). Early studies have indicated favorable results, and in some patients, symptom relief can occur within hours or days—rather than weeks (8, 9). Furthermore, recent studies on esketamine have gone beyond randomized clinical trials to include long-term, real-world, and observational study data. For instance, a real-world utilization study indicated that system and administrative-related factors, such as insurance coverage policies, prior authorization requirements, and site-of-care limitations, can influence both the availability and continued use of esketamine (11). Another observational real-world study conducted in routine clinical practice demonstrated clinically meaningful symptom improvement, with adverse event rates comparable to those reported in randomized controlled trials (12). Additionally, data from a long-term open-label extension study indicated that the therapeutic benefits of esketamine were maintained over time without any new or unexpected safety concerns (13). Post-approval safety analyses have further described the frequency of potential adverse effects, such as dissociation, sedation, and transient increases in blood pressure that reinforce the importance of closely monitoring esketamine-treated patients and carefully selecting suitable patients for esketamine therapy (14).

However, the introduction of esketamine to clinical practice has also raised some questions. Concerns about dissociative effects, the risk of misuse, and cost have all been noted by mental health practitioners (15–17). Logistical obstacles are also noteworthy. For instance, treatment rooms for esketamine nasal spray should include a comfortable reclining seat, be quiet with minimal distractions, potentially incorporate cognitive stimuli during the maintenance phase, have resuscitation equipment available for at-risk patients, and allow healthcare professionals easy access to specialized equipment while allowing them to remain close to the patient (18). Conversely, the rapid effect and potential for improvement in patients who have run out of options have garnered considerable interest (19, 20).

Esketamine use is still considered relatively emerging in Saudi Arabia, as it was registered by the Saudi Food and Drug Authority in 2021 (21). Further, little is known about how local psychiatrists view the role of esketamine in treating TRD. The Saudi National Mental Health Survey has provided valuable information about the prevalence of mood disorders (3), but targeted research into clinicians’ attitudes toward new interventions, such as esketamine, remains scarce. Considering the importance of this subject area, this study aims to evaluate psychiatrists’ attitudes toward esketamine as treatment for TRD, understand its perceived benefits and risks, and identify the important variables when making decisions regarding its use. With Saudi Arabia expanding its mental health infrastructure and moving toward evidence-based and personalized psychiatric care, this study is crucial to understanding the next steps on this topic.

## Materials and methods

### Study design, setting, and participants

This cross-sectional study was conducted across Saudi Arabia, targeting those currently practicing and in training in Saudi Arabia. The inclusion criteria consisted of all psychiatrists (consultants, registrars, senior registrars, and residents) currently practicing or training in Saudi Arabia. The exclusion criteria included medical students, medical interns, and individuals with communication barriers that prevent them from completing the study tool. Based on an estimated total population size of 1350 individuals, including psychiatrists and those in training in psychiatry, in the country (22), the required sample size was calculated to be 300 participants, using http://www.raosoft.com/samplesize.html, with a 5% margin of error and a 95% confidence level. A convenience sampling method was used to recruit the participants. Participants were reached and invited to participate in the study through the Saudi Commission for Health Specialties (SCFHS), which is the main regulatory body for the medical field in Saudi Arabia, and through WhatsApp groups involving the targeted population. Of note, the target population was initially estimated by the research team to be 1,350 psychiatrists and trainees based on previously available data (22). However, upon distributing the study tool through SCFHS, the total number of registered individuals meeting the inclusion criteria was found to be 1,701, and the survey was distributed to this comprehensive cohort. Among them, 324 individuals accessed the survey, and 223 completed the questionnaire, representing a response rate of 19.05% and a completion rate of 68.83%.

### Survey instruments

The study tool, which was developed by SurveyMonkey (https://www.surveymonkey.com) and distributed electronically, consisted of a questionnaire developed by the research team and the Physician Motivation Adoption Scale (PMA). Notably, data collection took place between the end of December 2024 and mid-May 2025.

The questionnaire, which was developed by the research team, assessed sociodemographic and other related factors. It sought to extract socio-demographic information (such as age, gender, practice setting, and professional title) and information on the perspectives, awareness, usage, and perceived benefits and risks of esketamine. The questionnaire also contained a section about implementation and access to assess for the availability, cost, and logistical barriers to esketamine use. It also contained a section about the overall attitudes (perceptions of safety, efficacy, and applicability) of esketamine in clinical practice. Specifically, the research team developed the questionnaire based on what they found to be relevant and significant on the topic in the literature (23–30), as well as information the research team deemed to be important to explore.

The PMA is a validated 27-item scale measuring six dimensions of motivation: Functional, Conformity, Power, Hedonic, Patient Benefit, and Cognitive (31). Responses are rated on a 5-point Likert scale (1 = “strongly disagree” to 5 = “strongly agree”) (31). Cronbach’s alpha for each dimension ranges from 0.75 to 0.94, indicating strong internal consistency (31). This study used the modified version of the PMA adapted by Brendle et al. (2024) to specifically evaluate motivation to adopt esketamine nasal spray for TRD (20), noting that permission to use the modified PMA was obtained from the original authors.

### Ethical considerations

The study tool included a cover page outlining the study’s title, purpose, and the voluntary nature of participation. Anonymity and confidentiality were assured. Participants provided informed consent by clicking “Next” to proceed with the survey. The study was approved by the institutional review board at the College of Medicine at King Saud University (Research Project No. E-24-9384).

### Statistical data analysis

R Software version 4.4.0 (R Foundation for Statistical Computing, Vienna, Austria) was used to conduct statistical analyses. Cronbach’s alpha coefficient was used to measure the internal consistency of the questionnaire scales in a bid to assess their reliability; an alpha of 0.7 or higher was deemed satisfactory. Continuous variables were displayed as mean ± standard deviation (SD) or median (IQR). The Shapiro test and visual examination of Q-Q plots were used to determine the normality in continuous variables, and homogeneity of variance was evaluated using Levene’s test. Bivariate analysis was conducted using the Mann–Whitney test/Kruskal–Wallis test because the normality assumptions were unmet. Frequency (%) was used to represent categorical variables. The difference between proportions was assessed using the Freeman–Halton exact test/chi-squared tests. Ordinal logistic regression analysis was used to determine the relationship between various factors and a willingness to prescribe esketamine among psychiatrists (“Would you consider prescribing or recommending esketamine as part of a treatment plan for TRD?”). A two-tailed P-value of less than 0.05 was deemed statistically significant. Notably, a *post-hoc* power analysis indicated that the sample size (n = 223) provided approximately 82% power to detect effect sizes in bivariate analyses of the primary outcome, including associations with professional classification and prior esketamine prescribing experience.

## Results

### Demographics of surveyed physicians

**Figure 1:**
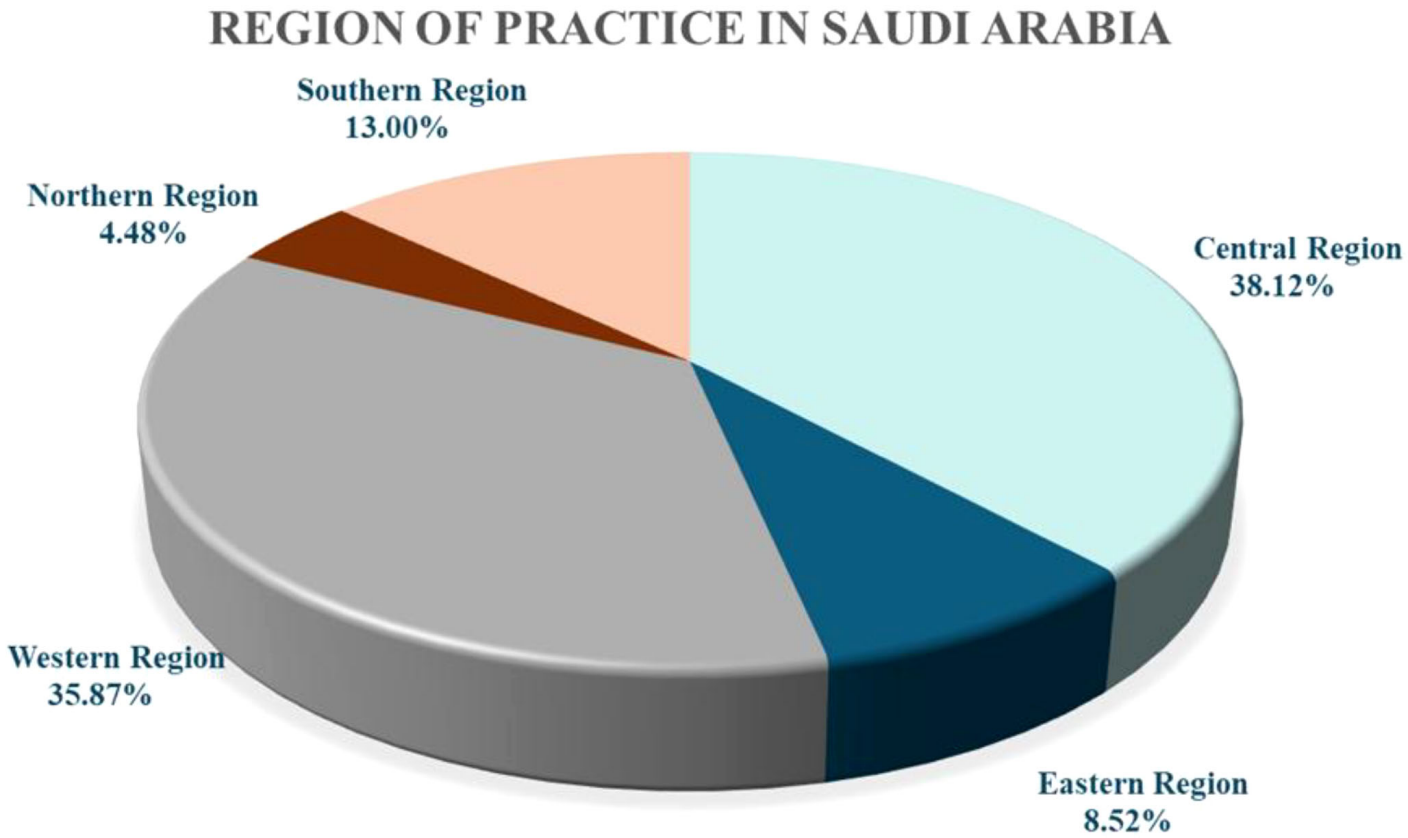
Regional distribution of psychiatrists’ practice.

**Table 1** lists the demographics of surveyed physicians. A total of 223 mental health professionals were enrolled in the study, with the majority being male (n = 152; 68.16%). It was found that more than two-thirds of physicians (n = 155; 69.51%) fell within the 23–35 years age group, and 37 respondents (16.59%) fell within the 36–45 years age group. Regarding the SCFHS classification, nearly half of the study participants (n = 105; 47.08%) were residents, and 53 (23.77%) were consultants. To evaluate representativeness, these figures were compared with those in the Ministry of Health (MOH) 2021 Statistical Yearbook, which reports a national total of 1,350 psychiatric providers (340 residents, 574 registrars, and 436 consultants) (22). While our sample somewhat aligns with the national distribution of consultants (23.77% in this study vs. 32.3% nationally), it features a relatively higher proportion of residents (47.08% vs. 25.2% nationally). This skew toward early-career physicians may reflect a stronger tendency among residents to engage in research and survey-based data collection. In terms of practice setting, the majority of participants (n = 190; 85.20%) worked in the governmental sector, 85 physicians (38.12%) worked in the central region of Saudi Arabia, and 80 physicians (35.88%) worked in the western region. The baseline demographics are presented in **Table 1**. Regional distribution of psychiatrists’ practice is illustrated in **Figure 1**.

**Table 1:** Demographics of surveyed physicians (*N* = 223).

Variable	Number (*n*)	Percentage (%)
Gender		
Male	152	68.16%
Female	71	31.84%
Age group		
23–35 years	155	69.51%
36–45 years	37	16.59%
46–55 years	16	7.17%
≥ 56 years	15	6.73%
SCFHS Classification		
Consultant	53	23.77%
Senior registrar	52	23.32%
Registrar	13	5.83%
Resident	105	47.08%
Primary Setting of Practice		
Private sector	13	5.83%
Government sector	190	85.20%
Both	20	8.97%
Region of practice in Saudi Arabia		
Central Region	85	38.12%
Eastern Region	19	8.52%
Western Region	80	35.88%
Northern Region	10	4.48
Southern Region	29	13.00%

### Clinical perspectives on esketamine

The study also explored the psychiatrists’ clinical perspectives on esketamine, **Table 2**. Among respondents, 44 (19.73%) reported ever prescribing esketamine in their practices. Of the 44 respondents, 21 (47.73%) had treated 1–2 patients with esketamine. Regarding the common/significant side effects, the participants thought esketamine was associated with dissociation/delusions/hallucinations (n = 149; 66.82%), followed by dizziness/vertigo (n = 132; 59.19%). Most psychiatrists were either slightly concerned/not concerned (n = 89; 39.91%) or moderately concerned (n = 87; 39.01%) about the potential of esketamine misuse or addiction. About 90 participants (40.36%) thought access to esketamine treatment was difficult in their region. Approximately two-thirds (n = 145; 65.02%) thought the cost of esketamine could be a significant barrier to prescribing it, and 148 (66.37%) thought the administration process of esketamine could be a significant barrier to prescribing it.

**Table 2:** Clinical perspective of esketamine (*N* = 223).

Concern	Number (*n*)	Percentage (%)
Have you ever prescribed esketamine	44	19.73%
How many patients have you treated with esketamine?	44	
1–2 patients	21	47.73%
3–5 patients	12	27.27%
6–10 patients	6	13.64%
11–20 patients	2	4.54%
21 patients or more	3	6.82%
What are the common/significant side effects you think esketamine might be associated with? ^#^		
Abuse and misuse	118	52.91%
Dissociation/delusions/hallucinations	149	66.82%
Nausea/vomiting/headache	131	58.74%
Dizziness/vertigo	132	59.19%
Lethargy	48	21.52%
Increased blood pressure	122	54.71%
Dysgeusia (altered taste)	39	17.49%
Euphoric mood/anxiety	94	42.15%
Others	5	2.24%
How concerned are you about the potential of esketamine misuse or addiction?		
Extremely concerned/very concerned	26	11.66%
Moderately concerned	87	39.01%
Slightly concerned/not concerned	89	39.91%
I am not sure	21	9.42%
How easy do you think the access to esketamine treatment is in your region?		
Easy	40	17.94%
Difficult	90	40.36%
Very difficult	58	26.01%
am not sure	35	15.69%
Do you think that the administration process of esketamine (including the need for certain preparation and monitoring) could be a significant barrier to prescribing it?		
Yes, I think it could be a significant barrier	148	66.37%
No, I do not think so	64	28.70%
I am not sure	11	4.93%

^#^More than one answer was allowed.

### Overall opinions of psychiatrists toward esketamine

**Table 3** presents the overall opinions of psychiatrists regarding esketamine. The most common causes that would make psychiatrists feel hesitant to prescribe esketamine were the administration process (n = 123; 55.16%), availability (n = 113; 50.67%), cost (n = 110; 49.33%), inexperience in prescribing it (n = 99; 44.39%), and safety/side effects (n = 76; 34.08%). Among the psychiatrists (n = 16) who reported other causes, 100% (n = 16) reported efficacy or evidence-related concern. Additionally, 170 physicians (76.23%) believed esketamine should be used/considered in patients with TRD. Most psychiatrists either probably (n = 97; 43.50%) or definitely (n = 80; 35.87%) would consider prescribing or recommending esketamine as part of a treatment plan for TRD. Among the study participants, 110 physicians (49.33%) might have considered prescribing esketamine to treat both TRD and suicidality. About three-fourths (n = 160; 71.75%) would rate esketamine as safe/somewhat safe overall, regardless of whether they had prescribed esketamine or not before. The most common factors psychiatrists thought would lead patients to be hesitant to accept esketamine were the administration process (n = 137; 61.43%), cost (n = 128; 57.40%), safety/side effects (n = 124; 55.60%), and availability (n = 87; 39.01%).

**Table 3:** Overall opinions of psychiatrists toward esketamine (*N* = 223).

Overall opinions toward esketamine	Number (*n*)	Percentage (%)
Which of the following would make you feel hesitant to prescribe esketamine?^#^	223	
Its availability	113	50.67%
Its cost	110	49.33%
Its safety/side effects	76	34.08%
Its administration process	123	55.16%
Not having experience in prescribing it	99	44.39%
Others	16	7.17%
Other causes ^#^	16	
Efficacy and/or evidence-related concern	16	100%
Patient selection-related concern	6	37.5%
Presence of alternative	3	18.75%
Prescribing-related concern	1	6.25%
Do you believe esketamine should be used/considered in patients with TRD		
Yes, I do	170	76.23%
No, I do not	20	8.97%
I am not sure	33	14.80%
Would you consider prescribing or recommending esketamine as part of a treatment plan for TRD?		
Yes, I definitely would	80	35.87%
Yes, I probably would	97	43.50%
No, I probably would not	25	11.21%
No, I definitely would not	2	0.90%
I am not sure	19	8.52%
For what indications might you consider prescribing esketamine?		
TRD	92	41.25%
Suicidality	7	3.14%
Both (TRD and suicidality)	110	49.33%
I am not sure	14	6.28%
Regardless of whether you prescribed esketamine or not before, how would you rate the overall safety of esketamine?		
Safe/somewhat safe	160	71.75%
Unsafe/somewhat unsafe	19	8.52%
I am not sure	44	19.73%
Which of the following factors do you think patients will be hesitant to accept taking esketamine due to?^#^		
Its availability	87	39.01%
Its cost	128	57.40%
Its safety/side effects	124	55.60
Its administration process	137	61.43%
Patient’s prior exposure to it	37	16.59%
Other (Novelty-related concern)	2	0.90%

^#^More than one answer was allowed. TRD, Treatment Resistant Depression.

### Participants’ responses to PMA items

**Figure 2:**
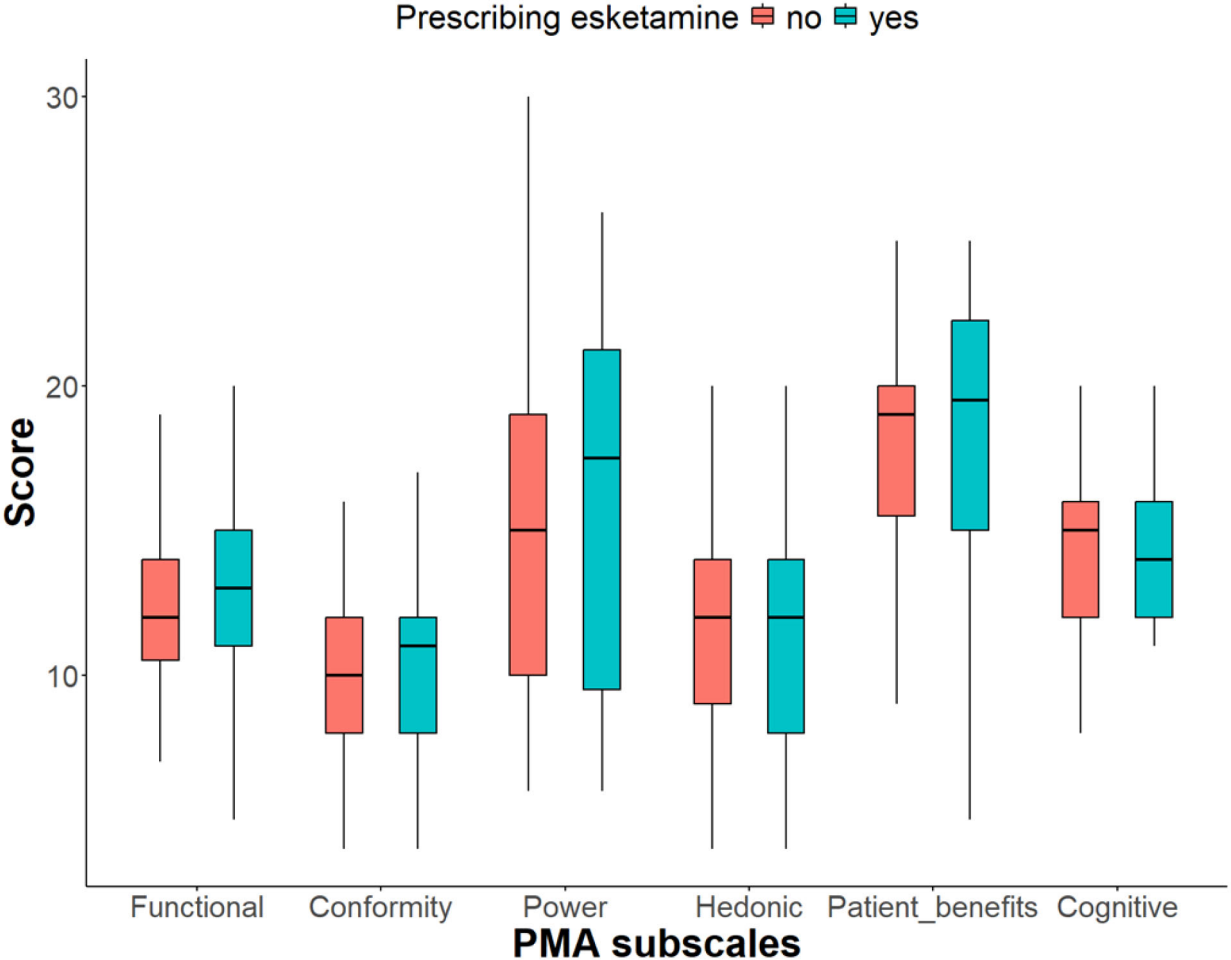
Scores of PMA subscales in prescribers vs. non-prescribers.

The PMA subscales (**Table 4**) had satisfactory internal consistency with Cronbach’s alphas > 0.7. It revealed that 77 (34.53%)–111 (49.78%) of respondents expressed neutrality regarding the adoption of esketamine in terms of reliability, effectiveness, practicability, and time-saving. Further, 90 (40.36%) and 80 (35.87%) disagreed concerning utilization and expectations; however, 109 (48.88%) and 80 (35.87%) of the respondents agreed neutrally with the majority opinion and advice. Similarly, 79 (35.43%)–65 (29.15%) of psychiatrists expressed neutral agreement on items related to impact on decision-making, power to achieve recognition, benefits of being a pioneer, becoming a key leader, and advancing careers. Additionally, 80 participants, corresponding to 35.87%, strongly disagreed with increasing their future earnings with esketamine adoption. Regarding hedonics, or enjoyment of the adoption of esketamine, 82 (36.77%) expressed excitement about acquiring medical innovations, whereas 71 (31.84%)–89 (39.91%) agreed neutrally with items related to passion, emotions, and fun. About patient benefits from esketamine, 81 (36.32%)–110 (49.33%) agreed to adopt esketamine if it convinced colleagues, increased the patients’ effort, time investment, learning efforts, and routine processes. Similarly, cognitive construct or having the understanding and knowledge to adopt esketamine had the highest scores, with 94 (42.15%)–113 (50.67%) thinking it improved skills, logical thinking, analysis, and intellectual challenge. The total scale revealed excellent internal consistency, with a Cronbach’s alpha of 0.90. **Figure 2** illustrates scores of PMA subscales in prescribers vs. non-prescribers.

**Table 4:** Participants’ responses to PMA Items (*n* = 223).

PMA subscales (dimensions)	Strongly disagree	Disagree	Neutral	Agree	Strongly agree	Sub-scale score mean ± SD (Min-Max)	Cronbach’s alpha
Functional						12.25 ± 3.22 (4-20)	0.81
F1 Reliability	16 (7.17%)	39 (17.49%)	111 (49.78%)	43 (19.28%)	14 (6.28%)		
F2 Time Saving	18 (8.07%)	30 (13.45%)	77 (34.53%)	80 (35.87%)	18 (8.07%)		
F3 Practicability	21 (9.42%)	71 (31.84%)	79 (35.43%)	42 (18.83%)	10 (4.48%)		
F4 Effectiveness	13 (5.83%)	31 (13.90%)	86 (38.57%)	72 (32.29%)	21 (9.42%)		
Conformity						9.61 ± 2.88 (4-17)	0.77
Con1 Expectations	52 (23.32%)	80 (35.87%)	64 (28.70%)	24 (10.76%)	3 (1.35%)		
Con2 Advice	43 (19.28%)	77 (34.53%)	80 (35.87%)	21 (9.42%)	2 (0.90%)		
Con3 Utilization	66 (29.60%)	90 (40.36%)	58 (26.01%)	6 (2.69%)	3 (1.35%)		
Con4 Majority opinion	23 (10.31%)	41 (18.39%)	109 (48.88%)	43 (19.28%)	7 (3.14%)		
Power						15.04 ± 6.02 (6-30)	0.94
P1 Recognition	49 (21.97%)	44 (19.73%)	77 (34.53%)	39 (17.49%)	14 (6.28%)		
P2 Career	44 (19.73%)	46 (20.63%)	65 (29.15%)	59 (26.46%)	9 (4.04%)		
P3 Opinion leader	51 (22.87%)	58 (26.01%)	70 (31.39%)	36 (16.14%)	8 (3.59%)		
P4 Decision makers	51 (22.87%)	49 (21.97%)	79 (35.43%)	37 (16.59%)	7 (3.14%)		
P5 Future earnings	80 (35.87%)	51 (22.87%)	57 (25.56%)	27 (12.11%)	8 (3.59%)		
P6 Pioneer	69 (30.94%)	52 (23.32%)	71 (31.84%)	25 (11.21%)	6 (2.69%)		
Hedonic						11.48 ± 3.75 (4-20)	0.89
H1 Passion	30 (13.45%)	55 (24.66%)	89 (39.91%)	41 (18.39%)	8 (3.59%)		
H2 Fun	35 (15.70%)	48 (21.52%)	71 (31.84%)	58 (26.01%)	11 (4.93%)		
H3 Emotions	36 (16.14%)	50 (22.42%)	79 (35.43%)	49 (21.97%)	9 (4.04%)		
H4 Excitement	23 (10.31%)	33 (14.80%)	69 (30.94%)	82 (36.77%)	16 (7.17%)		
Patient Benefit						18.36 ± 4.11 (5-25)	0.88
PB1 Increased effort	8 (3.59%)	5 (2.24%)	36 (16.14%)	110 (49.33%)	64 (28.70%)		
PB2 Time-consuming	6 (2.69%)	21 (9.42%)	70 (31.39%)	91 (40.81%)	35 (15.70%)		
PB3 Routine processes	9 (4.04%)	34 (15.25%)	64 (28.70%)	81 (36.32%)	35 (15.70%)		
PB4 Learning efforts	6 (2.69%)	17 (7.62%)	44 (19.73%)	87 (39.01%)	69 (30.94%)		
PB5 Convince colleagues	10 (4.48%)	20 (8.97%)	75 (33.63%)	81 (36.32%)	36 (16.14%)		
Cognitive						14.52 ± 3.21 (4-20)	0.89
Cog1 Analytical mind	10 (4.48%)	18 (8.07%)	80 (35.87%)	98 (43.95%)	17 (7.62%)		
Cog2 Intellectual challenge	12 (5.38%)	20 (8.97%)	69 (30.94%)	94 (42.15%)	28 (12.56%)		
Cog3 Improve skills	6 (2.69%)	3 (1.35%)	49 (21.97%)	113 (50.67%)	52 (23.32%)		
Cog4 Logical thinking	7 (3.14%)	3 (1.35%)	72 (32.29%)	100 (44.84%)	40 (17.94%)		

### Bivariate analysis: associations between prescribed esketamine and physician characteristics

Forty-four psychiatrists had prescribed esketamine, corresponding to a frequency of 19.73%. **Table 5** lists the association between various sociodemographic and other factors and prescribing esketamine as determined through bivariate analysis. There was a statistically significant difference between the frequency of prescribing esketamine and age, SCFHS classification, region of practice, concern about the potential of esketamine misuse or addiction, access to esketamine, and the administration process barrier. Further, 54.55% (n = 24) of psychiatrists who had prescribed esketamine were in the 23–35 age group, whereas 73.18% (n = 131) of psychiatrists who had not prescribed esketamine were in the same age group. Consultants represented a substantially higher proportion of prescribers (n = 18; 40.91%) than non-prescribers (n = 35; 19.55%); however, residents represented a higher proportion of non-prescribers (n = 94; 52.51%) than prescribers (n = 11; 25.00%). Psychiatrists who practiced in the central region were 43.58% (n = 78) among the non-prescribing group, compared to 15.91% (n = 7) among the prescribing group. Psychiatrists who had prescribed esketamine were slightly concerned/not concerned about the potential of esketamine misuse or addiction (n = 25; 56.82%), compared to 35.75% (n = 64) among the non-prescribing group. Psychiatrists thought that easy access to esketamine treatment in their region was 36.36% (n = 16) among psychiatrists who had prescribed it, compared to 13.41% (n = 24) among the non-prescribing group, whereas 29.55% (n = 13) of those who believed it had difficult access among the prescribing group, compared to 43.02% (n = 77) among the non-prescribing group. Further, 47.73% (n = 21) of psychiatrists who had prescribed esketamine thought the administration process was a significant barrier to prescribing it, compared to 70.95% (n = 127) among the non-prescribing group. Most psychiatrists who prescribed esketamine would rate esketamine as safe/somewhat safe (n = 39; 88.64%), compared to 67.60% (n = 121) among the non-prescribing group. There was no significant difference between psychiatrists who had prescribed esketamine and those who had not regarding gender, primary setting of practice, cost barrier, considering esketamine in patients with TRD, recommending esketamine as part of a treatment plan for TRD, and indications of esketamine.

**Table 5:** Relationship between prescribed esketamine and physician characteristics.

Physician characteristics	Prescribed esketamine N = 223		Test of significance	p-value
	No N = 179	Yes N = 44		
Age group (years)			Freeman–Halton exact test	0.049*
23-35	131 (73.18%)	24 (54.55%)		
36-45	28 (15.64%)	9 (20.45%)		
46-55	10 (5.59%)	6 (13.64%)		
≥56	10 (5.59%)	5 (11.36%)		
Gender				
Male	118 (65.92%)	34 (77.27%)	χ2 = 1.61	0.205
Female	61 (34.08%)	10 (22.73%)		
SCFHS classification			Freeman–Halton exact test	0.003**
Consultant	35 (19.55%)	18 (40.91%)		
Senior registrar	41 (22.91%)	11 (25%)		
Registrar	9 (5.03%)	4 (9.09%)		
Resident	94 (52.51%)	11 (25%)		
Primary Setting of Practice			Freeman–Halton exact test	0.630
Private sector	11 (6.15%)	2 (4.54%)		
Governmental sector	150 (83.80%)	40 (90.91%)		
Both	18 (10.06%)	2 (4.54%)		
Region of practice in Saudi Arabia			Freeman–Halton exact test	<0.001***
Central Region	78 (43.58%)	7 (15.91%)		
Eastern Region	14 (7.82%)	5 (11.36%)		
Western Region	64 (35.75%)	16 (36.36%)		
Northern Region	6 (3.35%)	4 (9.09%)		
Southern Region	17 (9.50%)	12 (27.27%)		
How concerned are you about the potential of esketamine misuse or addiction?			Freeman–Halton exact test	0.027*
Extremely concerned/very concerned	25 (13.97%)	1 (2.27%)		
Moderately concerned	73 (40.78%)	14 (31.82%)		
Slightly concerned/not concerned	64 (35.75%)	25 (56.82%)		
I am not sure	17 (9.50%)	4 (9.09%)		
How easy do you think access to esketamine treatment is in your region?			χ2 = 13.51	0.004**
Easy	24 (13.41%)	16 (36.36%)		
Difficult	77 (43.02%)	13 (29.55%)		
Very difficult	47 (26.26%)	11 (25.00%)		
I am not sure	31 (17.32%)	4 (9.09%)		
Do you think that the cost of esketamine could be a significant barrier to prescribing it?			χ2 = 0.69	0.707
Yes, I think it could be a significant barrier	117 (65.36%)	28 (63.64%)		
No, I do not think so	28 (15.64%)	9 (20.45%)		
I am not sure	34 (18.99%)	7 (15.91%)		
Do you think that the administration process of esketamine could be a significant barrier to prescribing it?			Freeman–Halton exact test	0.007**
Yes, I think it could be a significant barrier	127 (70.95%)	21 (47.73%)		
No, I do not think so	43 (24.02%)	21 (47.73%)		
I am not sure	9 (5.03%)	2 (4.55%)		
Do you believe esketamine should be used/considered in patients with TRD?			Freeman–Halton exact test	0.107
Yes, I do	131 (73.18%)	39 (88.64%)		
No, I do not	18 (10.06%)	2 (4.55%)		
I am not sure	30 (16.76%)	3 (6.82%)		
Would you consider prescribing or recommending esketamine as part of a treatment plan for TRD?			Freeman–Halton exact test	0.191
Yes, I definitely would	58 (32.40%)	22 (50.00%)		
Yes, I probably would	80 (44.69%)	17 (38.64%)		
No, I probably would not	21 (11.73%)	4 (9.09%)		
No, I definitely would not	2 (1.12%)	0		
I am not sure	18 (10.06%)	1 (2.27%)		
For what indications might you consider prescribing esketamine?			Freeman–Halton exact test	0.150
TRD	77 (43.02%)	15 (34.09%)		
Suicidality	7 (3.91%)	0		
Both (TRD and suicidality)	82 (45.81%)	28 (63.64%)		
I am not sure	13 (7.26%)	1 (2.27%)		
Regardless of whether you prescribed esketamine or not before, how would you rate the overall safety of esketamine?			Freeman–Halton exact test	0.020*
Safe/somewhat safe	121 (67.60%)	39 (88.64%)		
Unsafe/somewhat unsafe	18 (10.06%)	1 (2.27%)		
I am not sure	40 (22.35%)	4 (9.09%)		

χ2: chi-square test. *p < 0.05, **p < 0.01, ***p < 0.001.

### Associations between the PMA scale and physicians’ characteristics

**Table 6** lists the association between the PMA scale and physicians’ demographic characteristics. There is a statistically significant difference in the functional subscale score regarding age and SCFHS classification. There is a statistically significant difference in the conformity subscale score regarding age and region of practice in Saudi Arabia and in the power subscale score regarding SCFHS classification. Regarding the PMA scale total score, a statistically significant difference in SCFHS classification is observed.

**Table 6:** Relationship between PMA scale and physicians’ demographic characteristics.

	Functional	Conformity	Power	Hedonic	Patient benefits	Cognitive	Total
Age group (years)							
23-35	12 (10-14)	10 (8-12)	16 (11-19)	12 (9-15)	19 (15.5-21)	15 (12-16)	81 (73-91)
36-45	13 (12-14)	9 (6-10)	14 (6-18)	12 (10-14)	18 (14-20)	16 (13-17)	78 (70-89)
46-55	14 (12-16.25)	11.5 (9.75-12)	18 (12.75-20.5)	12 (8.75-12.5)	20 (15.75-21)	13 (12-15)	82 (75-97.25)
≥56	14 (11-14.5)	12 (10-13)	18 (13-21.5)	12 (10.5-13.5)	17 (16-20.5)	15 (13.5-16)	91 (76-96)
p-value	0.012*	0.004**	0.098	0.865	0.431	0.246	0.320
Gender							
Male	12 (11-14)	10 (8-12)	16 (10-19)	12 (8-14)	19 (15-21)	15 (12-16)	81 (72.75-92)
Female	12 (10.5-14)	10 (8-12)	14 (12-19)	12 (9.75-14)	19 (16-20)	16 (12-16)	81 (74-91)
p-value	0.456	0.987	0.968	0.608	0.730	0.356	0.904
SCFHS classification							
Consultant	12 (10-14)	9 (7-12)	13 (6-18)	12 (8-13)	19 (15-20)	16 (12-16)	78 (69-88)
Senior registrar	12.5 (11-14)	11 (8-12)	18 (13-19.25)	12 (10-14)	18 (16-20)	15.5 (12.75-17)	84 (77.75-93)
Registrar	15 (14-17)	11 (10-12)	18 (14-20)	14 (12-15)	17 (15-23)	14 (13-16)	88 (83-96)
Resident	12 (10-14)	10 (8-12)	14 (10-19)	12 (9-15)	19 (16-21)	15 (12-16)	81 (73-91)
p-value	0.004**	0.117	0.035*	0.061	0.333	0.909	0.021*
Primary Setting of Practice							
Private sector	14 (12-14)	9 (7-10)	16 (13-19)	10 (10-13)	17 (15-20)	14 (12-16)	81 (76-84)
Governmental sector	12 (10.25-14)	10 (8-12)	16 (10-19.75)	12 (9-14)	19 (16-21)	15 (12-16)	81 (73-93)
Both	12 (10.5-14)	10 (8.75-10)	14 (12-17.25)	12 (10-14)	16 (15-20)	15 (12-17)	78 (72.5-85)
p-value	0.550	0.539	0.688	0.922	0.082	0.797	0.446
Region of practice in Saudi Arabia							
Central Region	12 (11-14)	9 (8-12)	14 (10-18)	12 (8-14)	18 (15-20)	15 (12-16)	79 (71-85)
Eastern Region	12 (10.5-13.5)	7 (4.5-10)	12 (7.5-14)	12 (9-13.5)	20 (17.5-21.5)	16 (13-17.5)	77 (73-85)
Western Region	12 (10-14)	10 (8-12)	17 (12-19)	12 (10-14)	19 (16-21)	16 (13-16)	85 (73-93.25)
Northern Region	14.5 (12.25-16)	11 (8-12)	16 (12-24)	12.5(10-14)	19.5 (16.5-20)	15.5 (14.25-16)	89 (75.25-100.25)
Southern Region	12 (12-15)	11 (9-12)	18 (12-21)	12 (9-15)	18 (15-20)	13 (12-16)	82 (76-96)
p-value	0.271	0.048*	0.054	0.864	0.329	0.079	0.148

*p < 0.05, **p < 0.01.

**Table 7** lists the association between the PMA scale and physicians’ clinical perspectives. There is a statistically significant difference in the functional subscale score regarding the administration process barrier. In addition, there is a statistically significant difference in the patient benefits subscale score regarding the administration process barrier. Regarding the PMA scale total score, a statistically significant difference in the administration process barrier is observed.

**Table 7:** Relationship between PMA scale and physicians’ clinical perspectives.

	Functional	Conformity	Power	Hedonic	Patient benefits	Cognitive	Total
Have you ever prescribed esketamine							
Yes	13 (11-15)	11 (8-12)	17.5 (9.5-21.25)	12 (8-14)	19.5 (15-22.25)	14 (12-16)	83 (70.5-96.25)
No	12 (10.5-14)	10 (8-12)	15 (10-19)	12 (9-14)	19 (15.5-20)	15 (12-16)	81 (73-91)
P-value	0.247	0.148	0.239	0.815	0.706	0.357	0.362
How concerned are you about the potential of esketamine misuse or addiction?							
Extremely/very concerned	12 (11.25-13.75)	9.5 (7.25-12)	15.5 (12.25-18)	12 (10-14)	17 (15-20)	14.5 (12-16)	78.5 (74-87.75)
Moderately concerned	12 (10-14)	10 (8-12)	16 (11.5-19)	12 (9-14)	19 (15.5-20)	15 (12-16)	81 (73.5-91)
Slightly concerned/not concerned	12 (11-14)	10 (8-12)	16 (10-19)	12 (9-14)	19 (16-21)	16 (13-16)	81 (73-94)
I am not sure	12 (11-14)	10 (7-12)	16 (10-20)	12 (9-14)	18 (15-20)	13 (12-16)	78 (70-96)
P-value	0.936	0.892	0.956	0.957	0.237	0.122	0.633
Access barrier							
Easy	12 (10-15)	10 (8-12)	16 (11.5-20.25)	12 (9-15)	19 (16-20)	15 (12.75-17)	84 (75-95.25)
Difficult	12 (10.25-14)	10 (8-12)	17 (10.25-19)	12 (10-14)	19 (16-20)	15 (12-16)	81 (73-91)
Very difficult	12 (11-14)	9 (7-12)	13.5 (10-18.75)	12 (8-13)	18 (15-21)	15 (12-16)	79.5 (69.25-88)
I am not sure	12 (11-14)	10 (8-12)	16 (11.5-19.5)	12 (9-14)	20 (17-21)	16 (12.5-18)	81 (76-92.5)
P-value	0.849	0.736	0.784	0.704	0.608	0.530	0.391
Cost barrier							
Yes	12 (10-14)	10 (8-12)	17 (11-19)	12 (10-14)	19 (15-21)	15 (12-16)	81 (73-93)
No	12 (11-15)	9 (6-12)	13 (9-18)	10 (8-15)	19 (16-20)	15 (13-17)	80 (71-94)
I am not sure	13 (12-14)	9 (8-11)	14 (10-18)	12 (9-12)	19 (15-20)	15 (12-16)	78 (73-86)
p-value	0.393	0.224	0.107	0.301	0.738	0.704	0.468
Administration process barrier							
Yes	12 (10-14)	10 (8-12)	14 (9-19)	12 (8-14)	18 (15-20)	15.5 (12-16)	78.5 (71.75-89)
No	13 (11-15)	10 (8-12)	17 (12-21)	12 (9-14)	20 (17.75- 22.25)	15 (13-16)	85.5 (75.75- 96.25)
I am not sure	13 (12-14.5)	12 (10-12)	16 (12.5-19.5)	12 (10.5-12.5)	17 (15-20.5)	15 (13.5-15)	84 (77.5-90.5)
P-value	0.016*	0.189	0.183	0.830	0.031*	0.829	0.018*

*P < 0.05.

**Table 8** lists the association between the PMA scale and physicians’ opinions. There is a statistically significant difference in the functional subscale score regarding considering esketamine for patients with TRD, recommending esketamine as part of a treatment plan for TRD, considering indications of prescribing esketamine, and the overall safety of esketamine. There is a statistically significant difference in the power subscale score regarding recommending esketamine as part of a treatment plan for TRD. In addition, there is a statistically significant difference in the patient benefits subscale score regarding considering esketamine for patients with TRD and recommending esketamine as part of a treatment plan for TRD, and in the cognitive subscale score regarding recommending esketamine as part of a treatment plan for TRD. Regarding the PMA scale total score, there is a statistically significant difference in considering esketamine in patients with TRD, recommending esketamine as part of a treatment plan for TRD, and the overall safety of esketamine.

**Table 8:** Relationship between PMA scale and physicians’ opinions.

	Functional	Conformity	Power	Hedonic	Patient benefits	Cognitive	Total
Do you believe esketamine should be used/considered in patients with TRD?							
Yes	13 (12-14)	10 (8-12)	17 (10-20)	12 (9-14)	19 (16-21)	15 (12-16)	83 (74.25-94)
No	8 (7-10.25)	8.5 (7.5-12)	13.5 (6.75-18)	12 (9.75-12)	19.5 (15-21)	12 (12-16)	71.5 (65.25-79.5)
I am not sure	11 (9-12)	10 (8-12)	14 (12-18)	12 (9-14)	16 (15-20)	12 (12-16)	77 (73-85)
P-value	<0.001***	0.479	0.099	0.469	0.030*	0.138	<0.001***
Would you consider prescribing or recommending esketamine as part of a treatment plan for TRD?							
Yes, I definitely would	14 (12-16)	10 (8-12)	18 (10-22)	12 (9.75-15)	20 (17-23)	16 (12-17)	87.5 (78-98)
Yes, I probably would	12 (11-14)	10 (8-12)	15 (11-19)	12 (9-14)	19 (15-20)	15 (12-16)	81 (73-89)
No, I probably would not	10 (7-11)	9 (7-11)	13 (10-15)	11 (9-13)	18 (15-21)	13 (11-16)	75 (69-78)
No, I definitely would not	4.5 (4.25-4.75)	6 (5-7)	6 (6-6)	8 (6-10)	22.5 (21.25-23.75)	11 (10.5-11.5)	58 (55.5-60.5)
I am not sure	12 (10.5-12.5)	10 (8.5-12)	16 (12-18.5)	12 (9.5-13.5)	17 (15-19.5)	15 (12.5-16)	81 (73.5-86.5)
P-value	<0.001***	0.219	0.010*	0.482	0.037*	0.024*	<0.001***
For what indications might you consider prescribing esketamine?							
TRD	12 (10-14)	10 (8-12)	14.5 (12-19)	12 (9.75-14)	19 (16-20)	15.5 (13-16)	81 (75-91)
Suicidality	10 (8.5-11.5)	5 (5-10)	7 (6-15.5)	10 (7.5-15)	21 (18.5-21)	16 (14-16.5)	69 (63-84.5)
Both (TRD and suicidality)	13 (12-14.75)	10 (8-12)	17 (10-19)	12 (9-14)	19 (15-20)	15 (12-16)	81.5 (73-94)
I am not sure	11 (8.5-12)	11 (7.25-12)	14 (6.25-18)	12 (8.5-13.75)	16 (15-21.75)	13 (10.25-15.75)	76.5 (72.25-81)
P-value	0.001**	0.243	0.231	0.990	0.436	0.149	0.136
Regardless of whether you prescribed esketamine or not before, how would you rate the overall safety of esketamine?							
Safe/somewhat safe	13 (11-14)	10 (8-12)	16 (10-20)	12 (9-14)	19 (16-20.25)	16 (12-16)	82 (73-94)
Unsafe/somewhat unsafe	10 (8.5-12)	9 (7-11.5)	14 (9.5-18)	11 (8-12)	17 (14.5-20)	13 (10.5-15)	76 (68-81)
I am not sure	12 (11.75-13.25)	10 (8-12)	15.5 (12-19)	12 (9.75-14)	19 (15-21)	14 (12-16)	81 (74.5-88.25)
P-value	0.001**	0.653	0.372	0.054	0.577	0.058	0.030*

*p < 0.05, **p < 0.01, ***p < 0.001.

### Logistic regression analysis of characteristics associated with willingness to prescribe esketamine

The ordinal logistic regression model demonstrated an acceptable overall fit, as indicated by the likelihood ratio test (p < 0.001), with a Nagelkerke pseudo-R² of 0.47, suggesting moderate explanatory power. Multicollinearity was assessed using variance inflation factors (VIFs), which were calculated from a linear regression model that included the same set of explanatory variables. The results of the ordinal logistic regression model are listed in **Table 9**. Notably, senior registrars, registrars, and residents are significantly less likely to prescribe esketamine compared to consultants, with an OR of 0.21, 0.17, and 0.10, respectively. Psychiatrists who were not sure that the cost of esketamine could be a significant barrier to prescribing it are less likely to prescribe esketamine compared to those who did not think that the cost could be a significant barrier (OR = 0.32, 95% CI: 0.12-0.84, p = 0.022). On the contrary, psychiatrists with higher functional subscale scores are significantly associated with higher odds of prescribing Esketamine (OR = 1.62, 95% CI: 1.42-1.86, p < 0.001). No associations were found for age, gender, primary setting of practice, region of practice, access, administration process, and PMA subscales, except for the functional subscale.

**Table 9:** Predictors of willingness to prescribe esketamine among psychiatrists (ordinal logistic regression analysis).

Predictors	Multivariate		
	OR	95% CI	P-value
Age group (years) (vs. 23-35)			
36-45	0.49	0.16-1.56	0.231
46-55	0.64	0.14-3.0	0.563
≥56	0.49	0.10-2.28	0.350
Gender (vs. female)			
Male	0.83	0.45-1.54	0.564
SCFHS classification (vs. Consultant)			
Senior registrar	0.21	0.06-0.65	0.007**
Registrar	0.17	0.04-0.75	0.018*
Resident	0.10	0.03-0.32	<0.001***
Primary Setting of Practice (vs. Both)			
Private sector	0.76	0.17-3.73	0.280
Governmental sector	1.71	0.64-4.56	0.716
Region of practice in Saudi Arabia (vs. Central Region)			
Eastern Region	0.40	0.14-1.17	0.094
Western Region	0.60	0.31-1.18	0.142
Northern Region	1.09	0.23-5.68	0.919
Southern Region	0.66	0.25-1.80	0.416
Access barrier (vs. Easy)			
Difficult	1.96	0.81-4.68	0.132
Very difficult	1.31	0.51-3.38	0.572
I am not sure	0.32	0.12-3.01	0.852
Cost barrier (vs. no)			
Yes	0.64	0.27-1.47	0.294
I am not sure	0.32	0.12-0.84	0.022*
Administration process barrier (vs. no)			
Yes	0.94	0.47-1.87	0.870
I am not sure	0.29	0.08-1.10	0.068
Functional	1.62	1.42-1.86	<0.001***
Conformity	0.91	0.80-1.03	0.130
Power	1.37	0.97-1.11	0.296
Hedonic	0.97	0.88-1.08	0.542
Patient benefits	1.01	0.93-1.09	0.880
Cognitive	1.11	0.99-1.25	0.076

OR, odds ratio; CI, confidence interval; *p < 0.05, **p < 0.01, ***p < 0.001. The following variables were excluded from the model due to multicollinearity or non-significance: “Do you have someone (e.g., a relative or friend) who can drive you home from the hospital after you get your medication?” and “Would you feel stigmatized for using such a drug?”

## Discussion

In this study, we evaluated mental health professionals’ perspectives on the use of esketamine in depression and their motivation to adopt it in Saudi Arabia. Our results revealed that only about one-fifth (19.73%) of the surveyed participants reported ever prescribing esketamine. Moreover, in our study, almost half of the psychiatrists who had prescribed esketamine (47.73%) had only treated one or two patients. This pattern suggests that esketamine use is still in an exploratory or experimental phase for most practitioners in Saudi Arabia, instead of being a regular part of their usual clinical practice. As such, interventions to facilitate the adoption of esketamine in Saudi Arabia are warranted, particularly for those patients who are in need of it. Moreover, future research to explore the barriers hindering esketamine prescription in the Saudi context is warranted.

In our study, over three-quarters (77.09%) of participants were open to considering or recommending esketamine for TRD. The high percentage of professionals who would consider prescribing esketamine reflects a profound acknowledgement of its established clinical value in treating TRD (32, 33). Furthermore, multiple studies have demonstrated efficacy in managing TRD (8, 34, 35). In addition, continuing esketamine nasal spray along with oral antidepressants significantly delayed relapse in TRD patients who had achieved remission or responded to initial esketamine treatment (36). However, in our study, only (19.73%) had prescribed it thus far. This discrepancy between openness to prescribing and actually prescribing it among our participants reflects a translational gap, where theoretical support for a treatment does not necessarily translate into practical application. We hypothesize that this discrepancy could be related to systemic and logistical obstacles in the Saudi context, interfering with incorporation into clinical practice. Efforts should be directed toward creating supportive environments that facilitate the integration of esketamine into clinical practice, thereby allowing more patients to benefit from this novel treatment.

The data from the PMA scale in our study further support the discrepancy between openness to prescribing and actually prescribing it among our participants. Psychiatrists were highly motivated by intellectual challenges and improving their skills, but they were neutral or did not agree on how practical it was in terms of saving time and being efficient. Similarly, in the United States, psychiatrists generally consider esketamine an effective treatment for TRD, but they believe that there are significant barriers to its access and use (20). This highlights a gap between acknowledging the medication’s theoretical worth and the challenges of putting it into practice. It is important to address the barriers to esketamine adoption despite the high interest from mental health professionals. Future initiatives should focus on developing strategies to improve the feasibility of esketamine administration and overcoming logistical challenges.

Furthermore, in our study, the administration process of esketamine was identified by the participants as the single greatest reason for hesitation among participants (55.16%) and the top perceived barrier for patients (61.43%). The administration process is also considered a barrier in the United States (11, 37). This concern is rooted in the regulated nature of esketamine’s distribution. Administration often requires dedicated observation time and space (18). This clearly poses a substantial logistical burden on busy clinical settings in our study. As most of our study participants worked in the governmental sector (85.20%), the need for a dedicated observation space and two-hour monitoring periods could represent a substantial burden on high-volume public clinical settings. Our finding that the administration process is a primary barrier also indicates a lack of institutional readiness to support this therapy’s requirements in Saudi Arabia. However, the bivariate analysis demonstrated that 70.95% of non-prescribers viewed the administration requirements (monitoring and dedicated space) as a significant barrier, compared to only 47.73% of active prescribers, suggesting that the perceived complexity of the protocol is a greater hurdle than the actual practice. In addition, one critical logistical requirement for esketamine administration is that patients must not drive for 24 h (38). This requirement necessitates a reliance on a family member or significant other for transportation, representing a significant challenge in Saudi culture, where mental illnesses carry significant stigma, and individuals might prioritize privacy regarding psychiatric care. Consequently, patients may refuse treatment, and psychiatrists may hesitate to recommend a therapy that requires repeated hospital visits with a companion. Furthermore, the non-prescribing group reported much higher levels of concern regarding the administration process as a significant barrier (70.95%) compared to those who had successfully integrated it into their practice (47.73%). This finding suggests a “familiarity bias,” where actual experience with the administration protocol reduces the perceived difficulty of the task. As such, efforts should focus on developing clear infrastructure to support the safe and efficient integration of esketamine into standard practice in Saudi Arabia, moving it beyond an exploratory phase toward a more established treatment option for TRD. Moreover, establishing dedicated “Esketamine Suites” within governmental hospitals to provide the necessary monitoring space and resuscitation equipment would also alleviate this logistical concern identified by non-prescribers.

Moreover, our study revealed that the cost and availability of esketamine are also perceived as significant barriers (65.02% and 66.37%, respectively). Our results reflect that cost limits its accessibility in Saudi Arabia. A single nasal spray bottle of esketamine costs around 953 Saudi Riyals (39). In the United States, the estimated cost for a two-month treatment ranges between 26,550 SAR and 39,825 SAR ($7080–$10620). Notably, this two-month cost approximation does not include physician, facility, and other non-medication charges (25). The high cost is not merely a patient-related barrier; it also presents a challenge to institutions in a public sector-dominated healthcare system, either through direct costs or complex reimbursement policies. Furthermore, the “not sure” response regarding cost as a barrier, which represents (18.39%) of the responses in our study, being negatively associated with willingness to prescribe may indicate a fear of the unknown financial implications or a lack of clear institutional policies, leading to hesitation among prescribers. These findings highlight the need for clear and transparent information regarding esketamine’s financial implications and reimbursement processes to reduce prescribers’ hesitation. In addition, efforts should be made to optimize the supply chain and distribution of esketamine to ensure consistent availability, thereby enabling more equitable access to this important treatment for patients with TRD.

Participants in our study commonly cited perceived side effects, particularly dissociation/delusions/hallucinations (66.82%) and dizziness (59.19%). These concerns align with actual reported side effects in patients from various international studies (35, 40, 41). This suggests that these challenges are not unique to Saudi Arabia but are global concerns inherent to esketamine treatment. Further, our study demonstrates that psychiatrists who had prescribed esketamine were significantly more likely to view the drug as “safe or somewhat safe” (88.64%) compared to non-prescribers (67.60%). Due to the consistent concerns about esketamine side effects, future research should revolve around developing and evaluating specific strategies to mitigate these adverse events in clinical practice. Moreover, these findings highlight the need to educate the patient comprehensively about potential side effects and apply monitoring protocols.

In our study, almost half of the physicians were “moderately concerned” (39.01%) or “slightly concerned/not concerned” (39.91%) about the potential for esketamine misuse or addiction. However, esketamine has demonstrated a good safety and tolerability profile, and real-world data confirm it does not appear to initiate substance abuse in at-risk populations (28). The discrepancy between clinical interest and actual adoption in Saudi Arabia is likely compounded by specific socio-cultural and structural dynamics. While international safety data are generally favorable, the moderate concern regarding esketamine’s potential for misuse must be contextualized within a society where religious and cultural norms strictly prohibit intoxicants. This cultural backdrop heightens institutional and professional sensitivity toward medications with perceived psychoactive properties or dependence risks, fostering a more cautious approach to adoption than might be seen in other regions. Future Saudi research should monitor long-term outcomes in diverse populations, including patients with a history of substance abuse to address any concerns. In addition, continuing strict administration protocols and implementing a nationwide registry for esketamine are crucial to ensure the appropriate use of esketamine.

Our findings reveal that esketamine adoption is significantly associated with physician seniority and age. While the majority of the total study sample (69.51%) consisted of younger physicians (aged 23–35), this group made up a much smaller proportion of actual prescribers (54.55%) compared to non-prescribers (73.18%). Conversely, consultants represented 40.91% of the prescribing group despite being only 23.77% of the total sample. This finding is likely due to existing regulations in Saudi Arabia that, to our knowledge, stipulate that esketamine must be prescribed by a psychiatry consultant. This regulatory requirement reinforces the idea that the adoption of high-risk, high-benefit medications is closely tied to professional experience, institutional support, and confidence, particularly within a system where such prescribing authority is concentrated at the consultant level. Notably, while some residents reported having prescribed the medication, this likely reflects their role within the structured residency training process rather than independent prescribing authority. In the Saudi training model, residents typically evaluate the patient and collaborate with the most responsible physician (MRP) to determine the treatment plan. Although the final legal responsibility and approval rest with the consultant, the resident’s involvement in the clinical discussion and the technical entry of the order account for the reported prescribing experience among trainees (42). Our findings on the effect of professional background and personal experience are consistent with those in Portuguese and Australian studies (32, 43). The findings highlight the importance of initiating structured training programs and mentorship programs for residents and registrars to build their confidence and become competent in prescribing esketamine. Moreover, it is essential to create institutional systems that facilitate the educational process from experienced consultants to those in training to ensure the adoption of novel therapies and enable patients to access advanced treatment for TRD.

## Strengths and limitations

This study has several notable strengths. This study is among the first studies addressing the topic of esketamine in the psychiatric field in Saudi Arabia. It engaged a large and diverse sample of practicing and training psychiatrists across various regions and settings in Saudi Arabia, increasing the possible generalizability of the findings. With 223 participants, the study captures a comprehensive scope of perspectives, including attitudes, prescribing behaviors, and perceived barriers. Additionally, the use of a validated and specifically adapted measurement tool, the PMA, provides a reliable framework for assessing motivational and attitudinal dimensions related to esketamine adoption. Finally, the findings offer practical implications for healthcare policy, specialized training, and pharmaceutical access within the Kingdom.

Certain limitations, however, should be acknowledged. The use of convenience sampling may have introduced selection bias; future studies could address this through random sampling to improve representativeness. The cross-sectional design limits causal inference, suggesting a need for longitudinal research to track attitudes over time. Few participants had experience in prescribing esketamine, which could have diminished statistical power and constrained the reliability of regression estimates. Consequently, the results on prescribing behavior ought to be viewed with caution and regarded as exploratory. Institutional policies, availability of esketamine services and monitoring resources, and regional variations in healthcare infrastructure were not explicitly accounted for, which may have impacted prescribing practices. This oversight potentially restricts the generalizability of the findings to all prescribers in Saudi Arabia. The over-representation of the Central region may also limit generalizability. Accordingly, multi-regional collaborative studies are warranted. Reliance on self-reported data raises concerns about recall and social desirability bias; future work could mitigate this by incorporating objective data sources, such as prescription databases. Additionally, including residents may introduce bias, as they lack independent prescribing authority; future research should utilize stratified sampling to isolate autonomous clinical perspectives. In addition, our focus on physician perspectives omits patient-level factors, such as patient preferences and outcomes; future research should integrate these perspectives while also utilizing qualitative interviews with clinicians to better explore the “why” behind evolving views and barriers to adoption as they gain more experience with the medication. In addition, although the survey was primarily distributed via the SCFHS mailing list, which includes all licensed psychiatrists in Saudi Arabia, the survey link was also shared within targeted WhatsApp groups as a supplementary communication method. While WhatsApp was used exclusively within closed professional groups, its use may have preferentially reached certain subgroups of psychiatrists (e.g., younger clinicians or those more actively engaged in digital messaging platforms). Nevertheless, primary distribution through the SCFHS mailing list supports broad coverage of the licensed psychiatrist population in Saudi Arabia. Furthermore, the PMA scale used in this study has not been formally validated or culturally adapted within the Saudi Arabian context. Due to the absence of a locally validated or translated version, the original English instrument was utilized. Although all participants were healthcare professionals with adequate proficiency in English, the lack of local psychometric validation may have implications for the validity and reliability of the findings, and certain cultural or healthcare system-specific factors may not have been fully captured. Future studies should undertake formal cultural adaptation and psychometric validation of this instrument before its broader application in the Saudi context.

## Conclusion

Our study aimed to understand the perceptions and motivations of Saudi psychiatrists regarding the adoption of esketamine for TRD. By identifying key facilitators and barriers, our findings offer insights to bridge the gap between interest and practical implementation of this novel therapy in the Saudi healthcare system. Despite showing significant openness to prescribing esketamine for TRD, mental health professionals in Saudi Arabia have been slow to adopt it, revealing a gap between interest and practice. This discrepancy is associated with logistical barriers, including a complex administration process that requires dedicated observation time and space, and financial challenges related to high costs and availability. Concerns about side effects and potential misuse might also contribute to prescriber hesitation. Addressing these obstacles is crucial for the integration of esketamine into routine clinical practice, thereby improving patient access to this important therapy. To improve the current situation, policymakers and healthcare administrators should develop dedicated treatment areas within existing facilities to streamline the administration process. Additionally, exploring various funding models, such as insurance coverage or government subsidies, could help mitigate the high-cost barrier. Moreover, it is important to continue strict administration protocols and establish a nationwide registry for esketamine, which would be crucial in ensuring its appropriate use. Implementing comprehensive, hands-on training programs and establishing mentorship initiatives led by experienced consultants would also empower residents and registrars, boosting their confidence and competence in prescribing esketamine.

## Data Availability

The raw data supporting the conclusions of this article will be made available by the authors, without undue reservation.
