# Supplementary material for: Mental health professionals’ perspective on the use of esketamine in treatment-resistant depression and their motivation to adopt it: a Saudi cross-sectional study
Source: Front Psychiatry. 2026 Feb 27;17:1726411. doi: 10.3389/fpsyt.2026.1726411 (PMC12982179; doi:10.3389/fpsyt.2026.1726411)
Supplement: Supplementary file 1 [file DataSheet1.pdf]

Supplementary Material

Study Tool; the Questionnaire Part

Section 1: Sociodemographic Information

Gender

- Male
- Female

Age

- 23–35 years
- 36–45 years
- 46–55 years
- 56 years or older

SCFHS classification

- Consultant
- Senior registrar
- Registrar
- Resident

Primary setting of practice

- Private sector
- Governmental sector
- Both

Region of practice in Saudi Arabia

- Central Region
- Eastern Region
- Western Region
- Northern Region
- Southern Region

Section 2: Clinical Perspectives on Esketamine

Have you ever prescribed esketamine?

- Yes
- No

How many patients have you treated with esketamine?

- 1–2 patients
- 3–5 patients
- 6–10 patients
- 11–20 patients
- 21 patients or more

What are the common/significant side effects you think esketamine might be associated with?

- Abuse and misuse
- Dissociation/delusions/hallucinations
- Nausea/vomiting/headache
- Dizziness/vertigo
- Lethargy
- Increased blood pressure
- Dysgeusia (altered taste)
- Euphoric mood/anxiety
- Other (please specify)

How concerned are you about the potential of esketamine misuse or addiction?

- Extremely concerned / very concerned
- Moderately concerned
- Slightly concerned / not concerned
- I am not sure

Section 3: Clinical Implementation and Access

How easy do you think access to esketamine treatment is in your region?

- Easy
- Difficult
- Very difficult
- I am not sure

Do you think that the cost of esketamine could be a significant barrier to prescribing it?

- Yes, I think it could be a significant barrier
- No, I do not think so
- I am not sure

Do you think that the administration process of esketamine (including the need for certain preparation and monitoring) could be a significant barrier to prescribing it?

- Yes, I think it could be a significant barrier
- No, I do not think so
- I am not sure

Section 4: Overall Opinions

Which of the following would make you feel hesitant to prescribe esketamine?

- Its availability
- Its cost
- Its safety/side effects
- Its administration process
- Not having experience in prescribing it
- Other (please specify)

Do you believe esketamine should be used/considered in patients with treatment-resistant depression (TRD)?

- Yes, I do
- No, I do not
- I am not sure

Would you consider prescribing or recommending esketamine as part of a treatment plan for TRD?

- Yes, I definitely would
- Yes, I probably would
- No, I probably would not
- No, I definitely would not
- I am not sure

For which indications might you consider prescribing esketamine?

- TRD
- Suicidality
- Both (TRD and suicidality)
- I am not sure

Regardless of whether you prescribed esketamine or not before, how would you rate the overall safety of esketamine?

- Safe / somewhat safe
- Unsafe / somewhat unsafe
- I am not sure

Which of the following factors do you think patients will be hesitant to accept taking esketamine due to?

- Its availability
- Its cost
- Its safety/side effects
- Its administration process
- Patient's prior exposure
- Other (please specify)
